# Saturation genome editing of *BARD1* resolves VUS and provides insight into BRCA1-BARD1 tumor suppression

**DOI:** 10.1101/2025.11.03.25339440

**Published:** 2025-11-06

**Authors:** Ivan Woo, Silvia Casadei, Matthew W. Snyder, Nahum T. Smith, Sabrina Best, Malvika Tejura, Pankhuri Gupta, Abbye E. McEwen, Mason Post, Audrey Hamm, Moez Dawood, Airi Hosokai, Alicia Xu, Riddhiman K. Garge, Shawn Fayer, Terra Brannan, Marcy E. Richardson, Sriram Pendyala, Sarah Heidl, Lara Muffley, Douglas M. Fowler, Lea M. Starita

**Affiliations:** 1 Brotman Baty Institute for Precision Medicine, Seattle, WA, USA; 2 Department of Genome Sciences, University of Washington, Seattle, WA, USA; 3 Division of Medical Genetics, University of Washington, Seattle, WA, USA; 4 Department of Laboratory Medicine and Pathology, University of Washington, Seattle, WA, USA; 5 Human Genome Sequencing Center, Baylor College of Medicine, Houston, TX USA; 6 Ambry Genetics, Aliso Viejo, CA, USA

## Abstract

BARD1 variants are associated with hereditary breast cancer and neuroblastoma, yet, 98% of missense variants remain variants of uncertain significance (VUS). We applied Saturation Genome Editing (SGE) to assess 8,818 SNVs and 2,097 3-base pair deletions for their effects on cell survival and RNA abundance. We found that 13% of missense variants are loss-of-function (LoF), with 98% in BARD1’s three folded domains. LoF missense variants in the ankyrin repeat and BRCT domains were enriched in breast cancer cohorts, linking their molecular functions to cancer risk. SGE discriminated known pathogenic from benign variants with exceptional accuracy (AUC = 0.99) and resolved 95.4% of VUS, demonstrating high clinical utility. The single nucleotide resolution allowed discrimination of variant effects on RNA abundance from those affecting protein function and provided further evidence linking BARD1’s function in homology directed repair to tumor suppression. This comprehensive variant effect dataset informs inherited cancer risk and treatment decisions.

## Introduction

The BRCA1 Associated RING Domain protein, BARD1 [OMIM 601593], is required for the BRCA1 tumor suppressor to fold and function^1–5^. *BARD1* variants have been also linked to hereditary breast cancer and more recently neuroblastoma through case-control studies^6,7^. Accordingly, *BARD1* is included on genetic testing panels for cancer risk. 3,725 unique single nucleotide variants (SNVs) and 29 3bp-deletions of *BARD1* have accumulated in ClinVar^8^. 2,289 (61.4%) SNVs and 19 (65.5%) 3bp-deletions are either classified as variants of uncertain significance (VUS) or have conflicting interpretations. For SNVs that cause missense amino acid changes, the overwhelming majority (2,077 of 2,126, 97.7%) are either VUS or have conflicting interpretations. Because of the moderate 17–30% lifetime risk for breast cancer^6^ conferred by germline pathogenic variants in *BARD1*, enhanced screening is recommended for heterozygous individuals^9^. Additionally, Poly (ADP-ribose) polymerase (PARP) inhibitors are FDA approved for BARD1-deficient breast cancers^10^ and have shown efficacy for neuroblastoma^11^. Thus, our inability to understand how missense variation affects BARD1 function, and classify variants accordingly, limits cancer risk assessment and treatment guidance for many individuals.

BARD1 has diverse molecular functions, mediated by interactions with multiple partners^1,12–15^. Most importantly, BARD1, like BRCA1, is required for repairing double-strand DNA breaks by homology-directed repair (HDR)^3,16,17^. BARD1 protein-protein interactions are largely mediated through its three folded domains: an N-terminal RING domain, a central ankyrin repeat domain (ARD), and C-terminal tandem BRCT repeat domain. The E3 ubiquitin ligase activity of the BRCA1-BARD1 RING domains promotes HDR through ubiquitylation of histone H2A, preventing binding of 53BP1 to sites of double-strand DNA breaks—thus promoting HDR vs. non-homologous end joining (NHEJ)^16^. The BRCT domain recognizes DNA damage-associated histone H2AK13ub/K15ub marks^16,18,19^ to recruit the BRCA1-BARD1 dimer to sites of DNA damage. The ARD recognizes unmethylated histone H4K20me0 tails marking newly replicated chromosomes^20^, thereby ensuring that HDR occurs only during the G2 phase of the cell cycle. While these and other interaction partners of BARD1 have been identified, we lack a granular understanding of the residues and specific interactions BARD1 requires to fulfill its role in HDR.

Comprehensive variant effect maps could resolve the need for functional data to interpret BARD1 VUS and refine our understanding of BARD1’s protein-protein interactions and functions. Powered by the revolution in DNA synthesis and sequencing, multiplexed assays of variant effect (MAVEs) can furnish such maps by measuring the functional impact of all possible single nucleotide or amino acid substitutions in a target gene^21,22^. By systematically assessing thousands of variants, MAVEs provide insights into sequence-function relationships, revealing key domains and residues required for protein function, delineating the roles of amino acids in protein stabilization versus protein interactions, uncovering previously unknown binding surfaces, and discerning the mechanism of disease-causing mutations.

**Figure 1.**
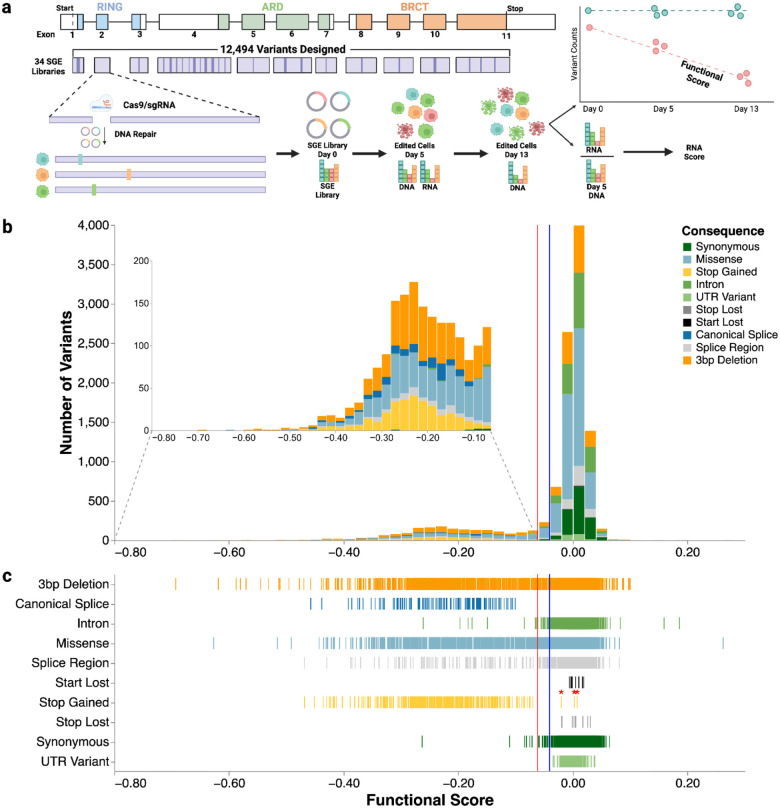
Saturation Genome Editing (SGE) of BARD1 generates variant effect scores for 10,915 variants (a) A map of the exon structure of *BARD1*, followed by the coverage of the 34 repair template libraries required to saturate all coding exons and proximal introns. Many exons that are too long for a single repair template library require sub-exon level repair template libraries and guide RNAs. For example, full coverage of exon 4 required sub-libraries A-L and 12 different guides. A schematic shows the SGE experimental strategy and scoring process. (b) A histogram of functional scores (n = 10,915). Variants are colored by mutational consequence as annotated by VEP^66^. The inset highlights LoF variants. (c) Strip plot of functional scores organized by mutational consequence (n = 10,915). The red vertical line indicates the threshold for the LoF class at −0.0615. Blue vertical line indicates the threshold for the functionally normal class at −0.0406

Variant effect maps also powerfully inform genomic medicine. The evidence provided by MAVE-derived functional data can drive the reclassification of more than half of VUS^23^. Saturation genome editing (SGE) is a type of MAVE where sequence variants are edited into the genome of a cell line, allowing each variant to be assessed for functional impact on cell survival^24–26^. The nucleotide resolution of SGE and endogenous genome editing provides insight into protein function while capturing the effect of variants on RNA expression and splicing. SGE of essential genes in haploid cells has been used to assess the effect of variants in *BRCA1*^25,27^, *DDX3X*^28^, *VHL*^29^, *BAP1*^30^, *BRCA2*^31^, and *RAD51C*^32^. These SGE data discriminate between pathogenic and benign variants with extremely high fidelity. BARD1, like BRCA1 and other members of the HDR pathway, is essential in HAP1 cells and is therefore amenable to SGE^33^ (Fig. 1a).

Here, we measured the effect of 8,818 *BARD1* SNVs and 2,097 3-base pair (bp) deletions on BARD1-dependent cell survival and the effect of 6,384 SNVs on BARD1 mRNA expression levels using SGE. LoF missense variants and deletions were enriched in the three folded domains relative to disordered domains. Our SGE data had near-perfect ability to discriminate between human pathogenic and benign variants, with an AUC = 0.99, and correctly identified the only bonafide pathogenic missense variant, p.Cys71Tyr as LoF. We showed that LoF missense variants in the ARD and BRCT domains are enriched in breast cancer cohorts, thereby linking the specific molecular functions of these domains to BARD1 tumor suppression in humans. Moreover, our data provided strong evidence for both pathogenic and benign germline variant classification, enabling resolution of 95.4% of VUS when functional evidence was applied. The nucleotide resolution of SGE enabled us to discern splice and RNA expression effects from protein effects, revealing that the annotated start codon is dispensable and that several synonymous and intronic variants are LoF. Analysis of missense variation across protein domains highlighted key residues for known protein interactions and identified a previously unknown interaction surface. Furthermore, while BARD1 has been ascribed multiple cellular roles, we provide additional evidence that its function in HDR is linked to cancer risk. In addition to unearthing new insights into BARD1’s tumor suppressor function, we provide a comprehensive variant effect dataset that can be used to inform inherited cancer risk and treatment decisions.

## Results

### Functional scores for 8,818 SNVs and 2,097 3-base pair deletions for *BARD1*

**Extended Data Figure 1.**
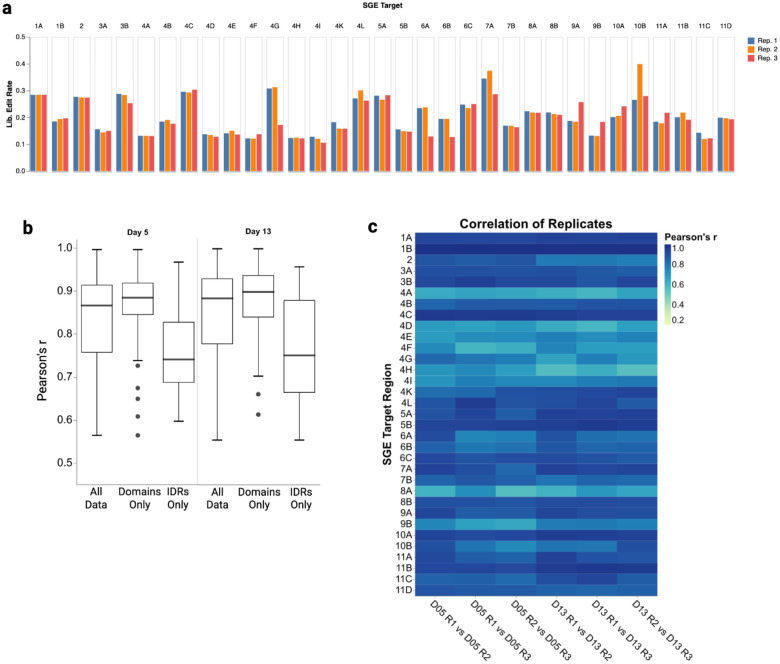
Editing rates and reproducibility across SGE experiments (a) Bar charts showing editing rates (Y-axis) generating usable reads across all QC-passing targets (Y-axis) and replicates (bar color). Usable reads are defined as having all fixed edits and a single programmed SNV or 3-bp deletion. (b) Box and whisker plots of median Pearson’s r correlation (Y-axis) across day 5 (left) and day 13 (right) timepoints. Data was subsetted as described on the X-axis. “All data” represents all data, “Domains Only” represents data only from the 3 structured domains, “IDRs Only” contains data from the exon 4-encoded disordered domain. (c) Heat map of Pearson’s r correlation between replicates (X-axis) for all QC-passing targets (Y-axis). Pearson’s correlation is colored for each target and replicate comparison

To perform SGE on all 2,334 bases of *BARD1*’s coding sequence and at least 10 bases of intronic or UTR sequence surrounding coding exons, we generated 34 sgRNAs and repair template libraries encoding all possible 9,351 SNVs and approximately 2,180 unique 3-bp deletions. For each of the 34 SGE regions, we transfected a sgRNA and matching repair template library into HAP1-LIG4Δ-Cas9 cells^28^ to induce editing by HDR. All editing experiments were performed in triplicate, with editing rates that generated usable sequences (*e.g*. having all required edits) ranging from 10.5% to 39.8% across SGE regions (Extended Data Fig. 1a). We PCR amplified and sequenced the edited SGE regions from genomic DNA at day 5 and day 13 to identify and count variants to calculate functional scores, and from mRNA at day 5 to calculate RNA scores. Variant counts were generally reproducible across individual replicates (median Pearson’s r = 0.87) (Extended Data Fig. 1b–c).

**Extended Data Figure 2.**
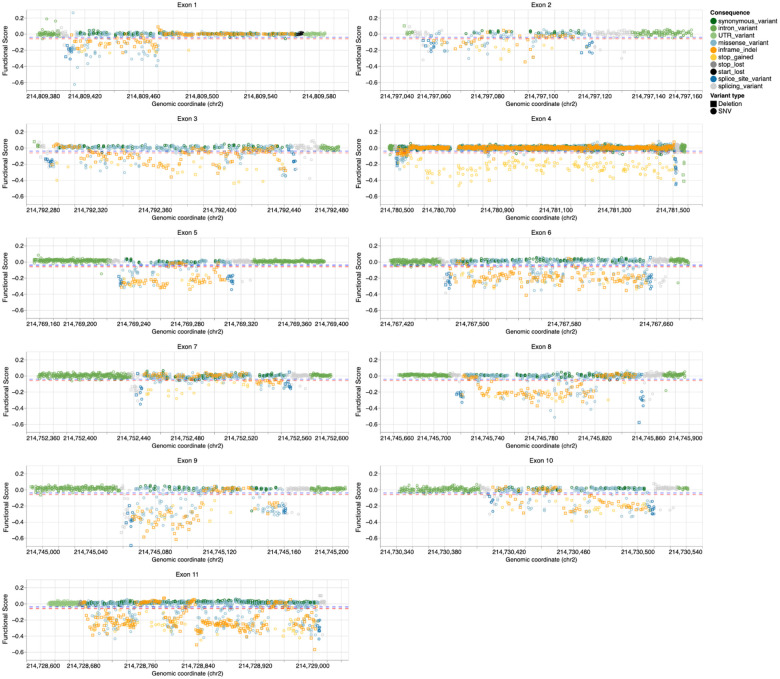
Functional scores for all SNVs and 3-bp deletions across *BARD1* exons Scatterplots of SGE score (Y-axis) vs. 1-based, hg38 genomic coordinates (X-axis) for each *BARD1* exon. SNV and inframe deletion variant types are represented by shape and molecular consequence of the variant is represented by color. Horizontal dotted red and blue lines represent score thresholds for classifying variants as LoF or functionally normal respectively.

**Extended Data Figure 3.**
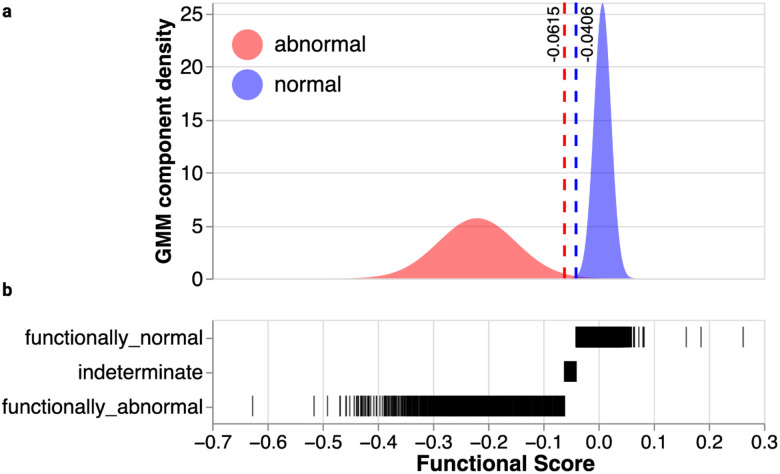
Fitted gaussians for variant functional classification (a) “Abnormal” (red) and “Normal” (blue) gaussians used to draw thresholds for classifying variants as LoF or functionally normal. Estimated density from GMM-modeling is on the Y-axis and SGE score is on the X-axis. Vertical red dashed line at X = −0.0615 represents upper threshold for LoF variants. Vertical blue dashed line at X = −0.0406 represents lower threshold for functionally normal variants. (b) Strip plot of functional class (Y-axis) vs. SGE score (X-axis)

We calculated functional scores—specifically, the estimated log_2_ fold change in relative variant abundance per day—for 95% of all possible SNVs (8,818) and 3-bp deletions (2,097), by applying a continuous-time linear regression model to adjusted log_2_ frequency ratios across all sampled time points and replicates (Fig. 1a, Extended Data Fig. 2 **and Supplementary File 1**). Synonymous and intronic variants, which mostly do not impact function, had scores near zero (Fig. 1b–c). Nonsense and canonical splice variants, which are expected to disrupt expression and protein function, were depleted from the cell population and had negative scores, confirming that BARD1 function is required for HAP1 cell survival. Most functional scores were near zero with a long tail of variants with negative scores that depleted over the course of the experiment. We used the distribution of synonymous/intronic variants and nonsense variants to determine thresholds to classify variants as functionally normal or loss-of-function (LoF) using a Gaussian Mixture Model (Fig. 1c and Extended Data Fig. 3). As expected, 365 of 368 (99.2%) nonsense variants were LoF and 1,286 of 1,306 (99.5%) synonymous variants were functionally normal. 94.4% of the 180 canonical splice variants were LoF. In sequence elements where some variants cause loss of function and others do not, such as splice regions, scores were more widely distributed, with 87.2% classified as functionally normal and 12.8% as LoF (Fig. 1c). Missense variant scores ranged widely, with 87.3% being functionally normal and 12.7% being LoF, suggesting that HAP1 cells are sensitive to loss of specific BARD1-dependent mechanisms required for cell survival.

### SGE reveals residues required for BARD1 function in the RING, ARD and BRCT domains

**Figure 2.**
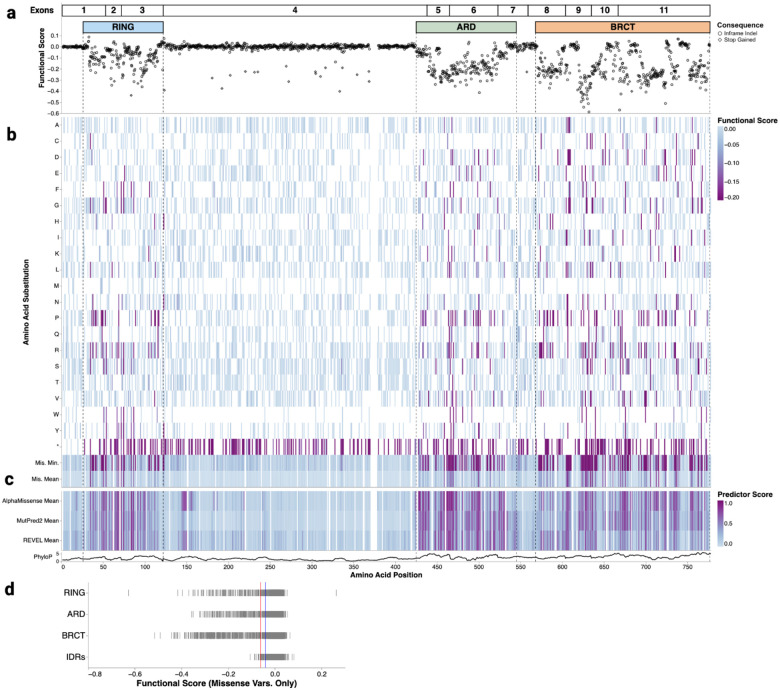
SGE identifies critical domains and functional residues of BARD1 (a) Map of programmed 3bp deletions (n = 2,097). Amino acid positions in BARD1 are on the X-axis and functional scores are on the Y-axis. In-frame deletions and deletions that cause stop-gain variants are denoted by shape. (b) A heatmap of functional scores for missense and stop-gained variants. Amino acid positions in BARD1 are on the X-axis and amino acid and stop-gained (*) changes are indicated on the Y-axis. Functional scores are colored as indicated. (c) Heatmap of minimum and mean functional scores across missense variants and mean variant effect predictor scores. For predictors, high scores denote predicted pathogenicity, while low functional scores indicate LoF. The bottom-most track is a line plot depicting the rolling mean phyloP score across a 10 amino acid residue window. (d) A strip plot of functional scores for missense variants grouped by protein domain. Red and blue vertical lines indicate functional class thresholds. IDRs denote the exon 4-encoded disordered domain.

BARD1 has three folded domains: an N-terminal RING domain, an ankyrin repeat domain (ARD), and a C-terminal tandem BRCT repeat domain. A large disordered domain encoded by exon 4 connects the RING and ARD, and a small disordered domain encoded by exons 7 and 8 connect the ARD and BRCT (Fig. 2a)^19,34^. We first validated that SGE scores reflect known functional effects by comparing them to results of previous orthogonal biochemical assays for HDR activity^35,36^, sensitivity to PARP inhibitors^16^, and *in vitro* ubiquitylation^18,37^ (Extended Data Fig. 5a). Functional scores were highly concordant with these assays, demonstrating that our cellular fitness readout integrates these known BARD1-dependent molecular and cellular functions.

**Extended Data Figure 4.**
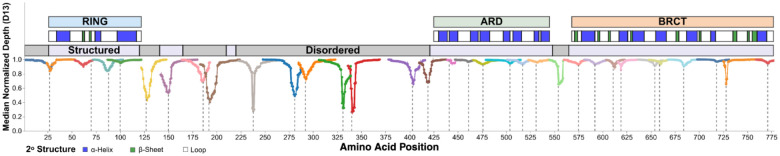
Median day 13 normalized read depth across *BARD1* Line plot of minimum median day 13 normalized depth (Y-axis) for all bases contributing to a single amino acid residue (X-axis) across the protein coding sequence of *BARD1*. Minima represent residues where sequencing reads contain deleted bases. Line color denotes different SGE targets. Vertical dashed gray lines denote CRISPR-Cas9 cut sites. Above the plot, the top-most track represents the BARD1 domains. The middle track represents secondary structure elements of each domain (white - loop, blue - alpha-helix, green - beta-sheet) as seen in solved structures for the RING (PDB 1JM7^54^), ARD (PDB 3C5R^69^), and BRCT (PDB 3FA2^67^). Bottom-most track depicts if the region is predicted to be structured (pLDDT > 50) (lavender) or unstructured (gray) by AlphaFold2^70^.

Within the exon 4-encoded disordered domain, only 2 of 20 depleting variants were 3bp deletions that did not introduce a premature stop codon (Fig. 2a). Furthermore, large, incidental in-frame deletions in disordered regions persisted over the course of the SGE experiments, whereas these deletions depleted in exons that encode folded domains (Extended Data Fig. 4). The ability of the disordered domains to tolerate both large incidental deletions and programmed in-frame deletions suggests that although the intrinsically disordered region encoded by exon 4 binds DNA and promotes DNA end resection^19,34^, very few, if any, single amino acids are likely to be necessary for critical BARD1 function. Moreover, of 1,802 missense SNVs scored in these regions, only 11 (0.61%) scored as LoF (Fig. 2b, d). Interestingly, one of the LoF missense variants, p.Lys140Asn has been shown to be important for BRCA1-BARD1-catalyzed end resection and RAD51 loading^38^.

A heatmap of functional scores across the BARD1 protein coding sequence shows that 98.1% (576 of 587) of LoF missense variants overlap the three folded domains: the RING, ARD and BRCT (Fig. 2b, d). This represents a 36-fold enrichment of LoF missense variants relative to the disordered domains (p = 2.8e-128, Fisher’s exact test). The RING, ARD, and BRCT were similarly sensitive to missense variation with 20.1%, 21.9%, and 23.5% of missense variants showing LoF respectively (Fig. 2b, d). Pairwise Fisher’s exact tests with Benjamini-Hochberg correction for multiple testing revealed no statistically significant differences (all adjusted p > 0.05). Furthermore, experimental replicates showed high correlation in the folded domains (median Pearson’s r = 0.89) suggesting robust score estimates for individual variants (**Figure S2**).

**Extended Data Figure 5.**
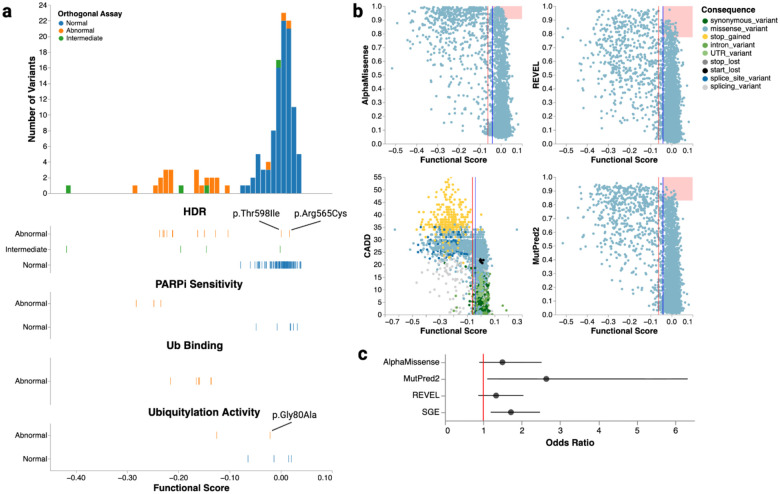
Comparison of BARD1 functional scores to variant effect predictors and orthogonal BARD1 functional assays (a) Histogram (top) and strip plots (bottom) of 125 variants assayed in orthogonal functional assays vs. SGE score (X-axis). In all plots, color represents the functional consequence of the variant in its respective assay. In the strip plots, plots are titled by the orthogonal assay used and the Y-axis represents the functional consequence of the variant. 3 variants that were functionally abnormal in orthogonal assays but functionally normal in SGE are highlighted. (b) Scatter plots comparing AlphaMissense^39^ (top-left), REVEL^41^ (top-right), CADD^42^ (bottom-left), and MutPred-2^40^ (bottom-right) to functional scores. Predictor scores are on the Y-axis and functional scores on the X-axis. Variants are colored by molecular consequence. Vertical red and blue lines represent SGE score thresholds for classifying variants as LoF or functionally normal respectively. Overlaid red rectangles highlight regions where predictors overpredict pathogenicity. Rectangles are drawn using the estimated score thresholds to yield moderate+ pathogenic evidence for each predictor^46^. (c) Odds ratios (X-axis) for occurrence of missense variants predicted to be LoF by predictors or SGE (Y-axis) in breast cancer cases vs. healthy controls. Results generated from the combined BRIDGES^6^ and CARRIERS^45^ population-based cohort. The vertical red line denotes an odds ratio of one. Points denote the estimated odds ratio and whiskers denote the 95% confidence interval.

Comparison of functional scores to AlphaMissense^39^, MutPred-2^40^, and REVEL^41^ missense variant effect predictions reveals similar patterns, with predicted LoF effects generally overlapping BARD1’s three folded domains (Fig. 2c). However, the predictors tended to overpredict pathogenicity (Fig. 2c and Extended Data Fig. 5a). Incorrectly predicted variants receiving at least moderate evidence towards pathogenicity were enriched in the ARD for all three predictors. Additionally, AlphaMissense predicted variants spanning residues 140 to 160 in the disordered domain to be pathogenic, but these variants generally had functionally normal functional scores (Fig. 2c). Similarly, the nucleotide-level variant effect predictor CADD^42^ predicted start-loss variants to be deleterious, but these had normal functional scores (Extended Data Fig. 5a).

### SGE reveals germline BARD1 variants that are linked to increased cancer risk

**Figure 3.**
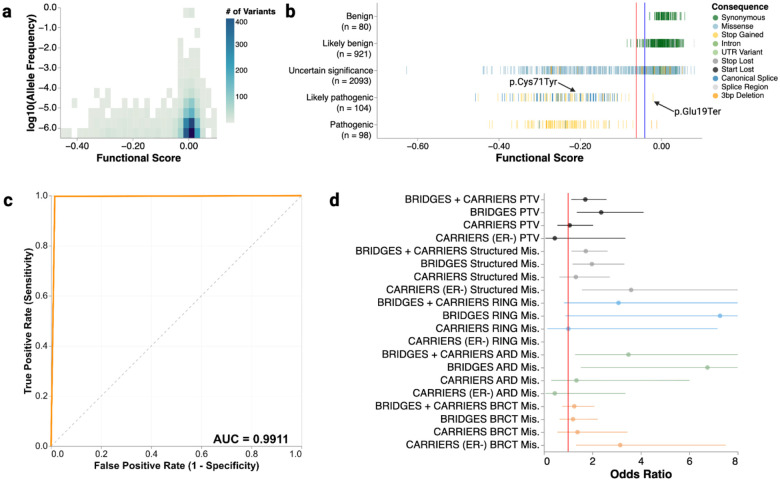
Comparison of BARD1 functional scores to human genetics studies (a) Heatmap of functional scores (X-axis) compared to log_10_-transformed minor allele frequency (MAF) (Y-axis) (n = 3,435). Variants were accessed from gnomAD^43^ and the Regeneron Million Exome Variant Browser^44^. Each bin is colored by the indicated number of variants in that bin. (b) A strip of BARD1 scores by ClinVar label (n = 3,296). All single nucleotide variants and 3-bp deletions accessed from ClinVar^8^ on September 12, 2025 and had at least 1-star review status. (c) Receiver operator characteristic (ROC) curve for the ability of BARD1 functional scores to discriminate pathogenic/likely pathogenic from benign/likely benign variants from ClinVar. (d) Odds ratios for the occurrence of LoF variants in breast cancer cases vs. controls in the listed cohorts for either all protein or specified domain. For all cohorts, only data from population-based studies was used. The vertical red line denotes an odds ratio of one. Points denote the estimated odds ratio and whiskers denote the 95% confidence interval.

Our SGE experiments were performed in a haploid human cell line. To determine whether the variant effects measured are relevant to human disease, we compared SGE results to human genetics databases. We expected that LoF BARD1 variants, like LoF BRCA1 variants, would have low minor allele frequency (MAF) in population databases^25^. We found that LoF BARD1 variants had significantly lower MAF than functionally normal variants in gnomAD^43^ (median MAF 6.2e-7 vs. 1.2e-6, p = 2.3e-7, Mann-Whitney U) and the Regeneron Million Exome Variant Browser^44^ (median MAF: 6.1e-7 vs. 1.2e-6, p = 0.001, Mann-Whitney U) (Fig. 3a). This suggests that variant effects measured by SGE are relevant to human health as variants not tolerated in SGE are also infrequent in the human population.

Functional scores of *BARD1* variants currently classified as pathogenic or likely pathogenic (PLP) and benign or likely benign (BLB) in ClinVar had different distributions (Fig. 3b). PLP variants had a mean functional score of −0.23 while BLB variants had a mean functional score centered around 0 (p = 6.4e-24, Student’s t-test). Of the variants scored by SGE, 202 variants are classified as PLP in ClinVar (139 stop-gained, 58 canonical splice, 1 missense, 4 other) and SGE correctly identified 199 (99%) as LoF. The single *BARD1* missense variant currently cataloged as likely pathogenic, p.Cys71Tyr, was correctly classified as LoF by SGE with a score of −0.21 (Fig. 3b). We correctly classified 987 of the 1,001 (99%) variants currently cataloged as BLB (711 synonymous, 134 splice-region, 40 missense, 116 other) as functionally normal with eleven variants falling in the indeterminate range. Thus, SGE showed high sensitivity and specificity for accurately identifying pathogenic and benign variants (AUC = 0.99, Fig. 3c) indicating that variant effect measurements generated by SGE are predictive of disease.

To further validate our results, we evaluated whether LoF missense variants were enriched in breast cancer cohorts. Leveraging the BRIDGES^6^ and CARRIERS^45^ breast cancer case control cohorts, we asked if LoF missense variants were more likely to be carried by individuals diagnosed with breast cancer relative to healthy controls. We found LoF missense variants in BARD1’s structured domains were enriched in breast cancer cases with an odds ratio of 1.73 (95% CI 1.14 – 2.63) (Fig. 3d) and all LoF missense BARD1 variants were enriched in breast cancer cases with an odds ratio of 1.72 (1.19 – 2.48) (**Supplementary Table 1**). Notably, the odds ratios for LoF missense variants (both all LoF missense and those only in the structured domains) were not significantly different from those of protein truncating variants (**Supplementary Table 1**) (p = 0.97 and p = 0.99 respectively, Z-test), suggesting that LoF missense and truncating *BARD1* variants confer similar cancer risk. Reinforcing the SGE results, missense variants identified as having at least moderate evidence of pathogenicity by the MutPred-2 variant effect predictor were also enriched in breast cancer cases with an odds ratio of 2.64 (95% CI 1.10 – 6.32) (Extended Data Fig. 5c)^40,46^. Moreover, in the CARRIERS cohort, breast cancer cases were annotated for estrogen receptor (ER) status. Subsetting for ER status, we found that germline LoF missense variants in BARD1’s structured domains were enriched in estrogen receptor negative (ER−) breast cancer cases with an odds ratio of 3.60 (95% CI = 1.58 – 8.23) compared to healthy controls (Fig. 3d), aligning well with the CARRIERS^45^ study where protein-truncating *BARD1* variants were associated with elevated ER− breast cancer risk.

While the only bonafide pathogenic *BARD1* missense variant, p.Cys71Tyr, is in the RING domain, we next investigated if LoF missense variants in the ARD and BRCT domains were also associated with increased disease risk. Most notably, we found that ARD LoF missense variants were enriched in the BRIDGES^6^ cohort (population-based OR = 6.75, 95% CI = 1.52 – 29.9, all sample cohorts OR = 7.07, 95% CI = 1.63 – 30.8) and LoF BRCT variants were also associated with increased ER− breast cancer risk in the CARRIERS^45^ cohort (OR = 3.15, 95% CI = 1.32 – 7.50) (Fig. 3d). Neither cohort had enough variants in the smaller RING domain for statistically significant results (**Supplementary Table 1**). Additionally, across BARD1, we did not find any statistically significant enrichment of missense variants that scored as functionally normal by SGE in breast cancer cases relative to healthy controls (**Supplementary Table 1**). Ultimately, our results demonstrate that the variant effect measurements generated by SGE are relevant to disease and that LoF missense variants across all three BARD1 functional domains are associated with increased cancer risk.

### BARD1 functional scores enable VUS reclassification

**Extended Data Figure 6.**
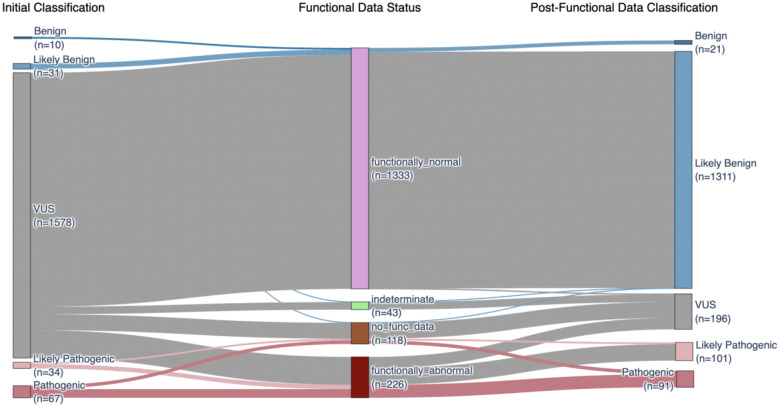
The clinical utility of data generated by *BARD1* SGE Sankey plot of all variants in Ambry Genetics’ database before and after application of functional data as indicated.

**Figure 4.**
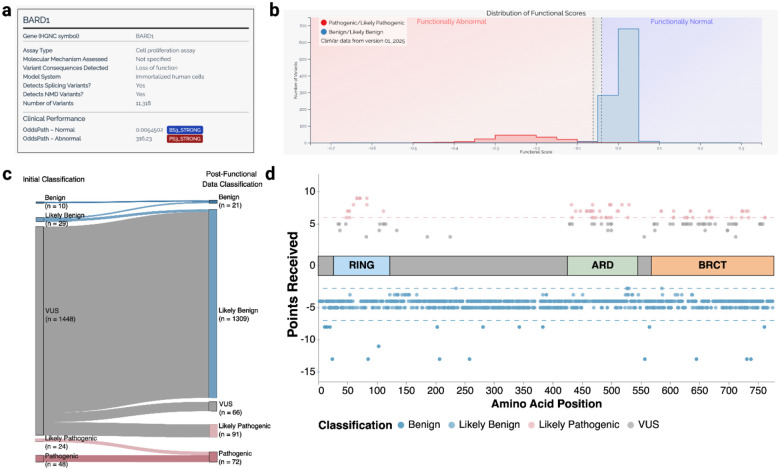
The clinical utility of functional evidence generated by BARD1 SGE (a) MaveMD Assay Fact Sheet for *BARD1* SGE. (b) MaveMD display of *BARD1* functional scores for clinical variants with thresholds used to define functional categories for the OddsPath calibration. (c) Sankey plot of Ambry VUS before and after addition of function evidence. (d) Scatter plot of missense variants reclassified using functional evidence. A cartoon of BARD1’s three domains is centered at Y = 0. Y-axis represents number points toward a benign (negative) or pathogenic (positive) classification. Horizontal lines denote the classifications thresholds; the pink line at Y = 6 denotes the threshold for “Likely Pathogenic”, the light blue line at Y = −2 denotes the cutoff for “Likely Benign”, and the blue line at Y = −7 denotes the cutoff for “Benign”. The X-axis represents the amino acid position of the variant. Variants are colored by their final classification.

Next, we assessed the clinical utility of these data for VUS reclassification. Calibration is required to translate functional scores to evidence for variant classification. The current ClinGen-approved calibration method calculates OddsPath, a Bayesian odds ratio used to assign evidence based on the ability of the assay to correctly identify previously classified pathogenic and benign variants as LoF or functionally normal, respectively^47^. We collected 186 PLP and 959 BLB variants from ClinVar for calibration (**Supplementary Table 2**). We included stop-gained, splice site and synonymous variants because the functional scores for nonsense variants overlapped LoF missense and synonymous overlapped functionally normal missense scores (Fig. 1c), LoF missense variants are linked to cancer risk through either family^48^ or case/control analyses, (Fig. 3d) and for practical purposes, there were too few classified pathogenic missense variants available to assign any benign evidence otherwise (Extended Data Fig. 6). The accurate positive and negative predictive values of SGE resulted in an OddsPath of 316.23 which equated to PS3_strong evidence (4 points) towards pathogenic classifications and an 1/OddsPath of 0.0054 for functionally normal, assigning strong evidence (−4 points) toward benign classifications (**Supplementary Table 2**). These calibration results for BARD1 can be visualized in MaveMD, a clinically focused display for the MaveDB database^49^ (Fig. 4a–b).

To reclassify VUS we combined the additional evidence from Ambry Genetics and the calibrated functional evidence to reclassify 87.6% (1,382/1,578) of VUS in Ambry’s database according to the ACMG/AMP v3 recommendations with the exception that likely benign classifications required −2 points^50,51^. For the 1,448 VUS for which we had functional data, 1,382 (95.4%) were reclassified. 1,291 (89.2%) VUS were reclassified as BLB and 91 (6.3%) are reclassified as PLP and 66 (4.5%) remain VUS (Fig. 4c, Extended Data Fig. 6, **and Supplementary Table 2**). The functional evidence enabled reclassification of all missense VUS receiving benign evidence. All remaining missense VUS have points toward pathogenic classifications, of those with +4 and +5 points (‘high VUS’), all but 1 are in BARD1’s 3 folded domains (Fig. 4d).

100% of reclassifications were driven by functional evidence. Computational predictions (PP3/BP4) contributed to 96% reclassifications, population frequency (PM2_supporting) contributed to 70%, and predicted functional consequence (PVS1) contributed to 0.6%. Variants that remained VUS did not have functional evidence (n = 88), had indeterminate functional scores (n = 42) or did not have enough evidence outside of functional data for reclassification (n = 66). The overall reclassification rate of 87.6% highlights the high clinical utility of the *BARD1* SGE data.

### SGE identifies SNVs that impact BARD1 expression

**Extended Data Figure 7.**
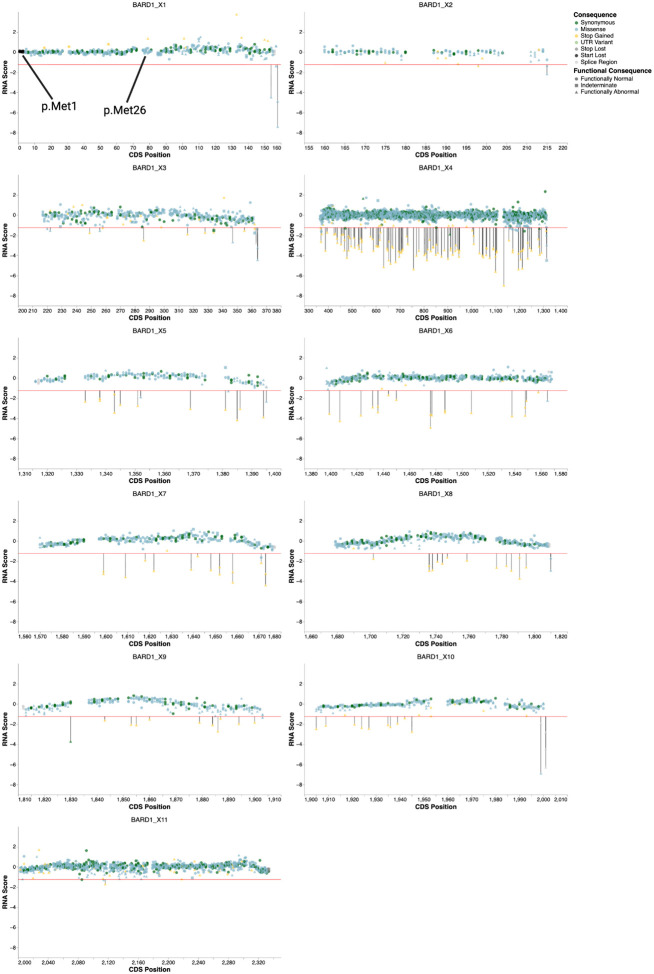
RNA scores for variants across *BARD1* Scatter plots of RNA score (Y-axis) vs. CDS position (X-axis) for all exons in *BARD1*. For all plots, the horizontal red line at Y = −1.24 represents the RNA score threshold used to classify variants as having “low” RNA abundance, representing one standard deviation above the mean RNA score for nonsense variants. Variants are colored by molecular consequence and shape denotes the SGE functional consequence. Variants classified as LoF with low RNA abundance are further highlighted with a black vertical line extending from the RNA threshold to the datapoint. In exon 1, variants stop-loss variants and variants impacting alternate start codon p.Met26 are highlighted.

**Figure 5.**
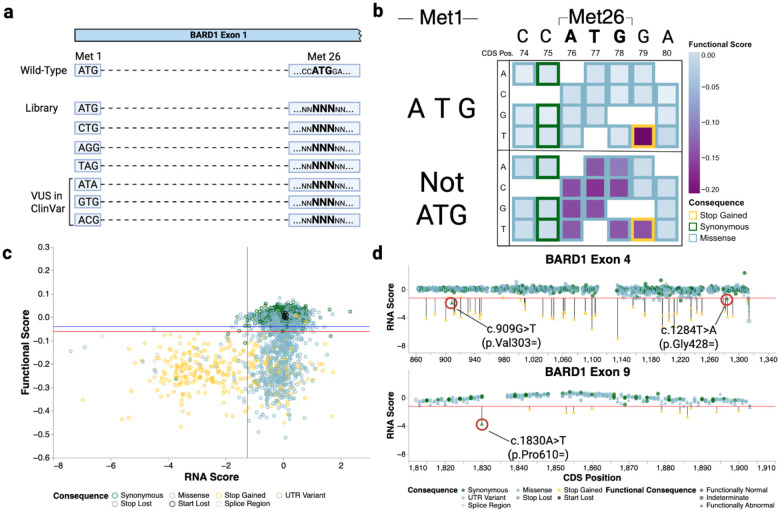
SGE identifies nucleotide variants that affect BARD1 expression and RNA abundance (a) Design of double mutant experiments to investigate start codon usage. A repair template library for exon 1A was designed such that each variant a p.Met26 was coupled to either the reference ATG at p.Met1 or seven other codons including variants currently listed in ClinVar. (b) Functional scores for double mutants experiments are displayed in nucleotide-level heatmaps grouped by whether p.Met1 was encoded by ATG or a variant codon. Each position in the coding sequence for p.Met26 and its surrounding bases are labeled on the X-axis. The nucleotide change is denoted on the Y-axis. Each box represents a SNV and the outline is colored by the mutational consequence, the fill color represents the SGE score (above) or average SGE score (below) for double mutants with variants at p.Met1. (c) Scatter plot of RNA scores (X-axis) vs. functional scores (Y-axis) (n = 6,384). Horizontal red and blue lines denote LoF and functionally normal SGE thresholds respectively. The vertical red line at X = −1.24 represents the RNA score threshold used to call variants depleted in the RNA. (d) Lollipop plots of exons 4 and 9 highlight synonymous changes that show LoF functional scores, depleted RNA scores, and are predicted to disrupt splicing. The red horizontal line represents the RNA score threshold for calling variants depleted in the RNA.

To parse LoF mechanisms, we first looked for unexpected results that might be explained by RNA- vs. protein-level variant effects. We noticed that start-loss variants and three stop-gained variants within the first exon were tolerated. Of the tolerated start-loss and stop-gained variants, three start-loss variants (p.Met1Ile, p.Met1Val, and p.Met1Thr) are VUS or have conflicting classifications of pathogenicity and one stop-gained variant (p.Glu19Ter) is PLP in ClinVar. The stop-gained variants occurred between the first annotated initiator codon and a methionine at position 26, suggesting p.Met26 might also be capable of initiating translation. Double mutant experiments targeting both initiators confirmed that both p.Met1 and p.Met26 can independently initiate BARD1 translation depending on context (Fig. 5a–b). When the first initiator was lost, variants abrogating the second initiator became deleterious. In addition we saw no loss of RNA expression for variants at either p.Met1 or p.Met26 (Extended Data Fig. 7). These results indicate that stop-gained variants upstream of p.Met26 and start-loss variants are unlikely to contribute to disease.

**Extended Data Figure 8.**
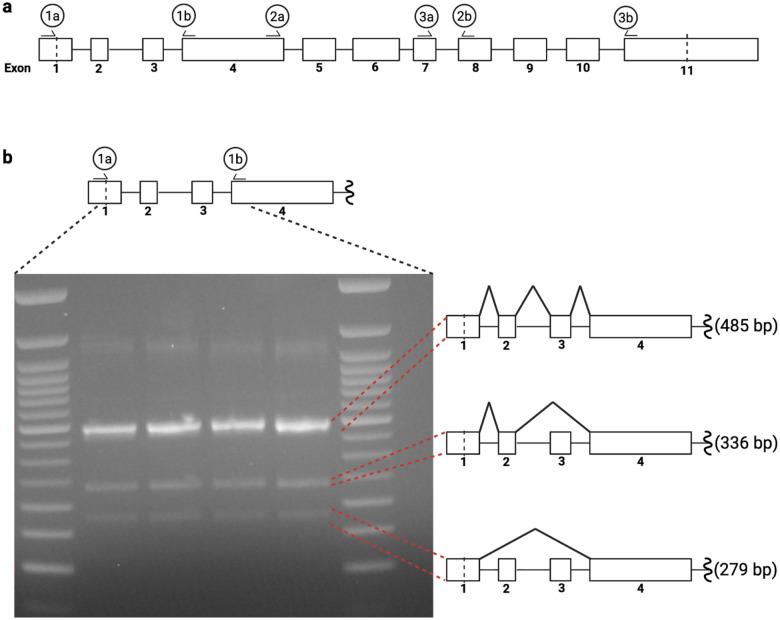
Alternate splicing of the *BARD1* transcript in HAP1 (a) Illustration of *BARD1* exons and primers used for RT-PCR to assess presence of alternate *BARD1* isoforms in HAP1. Large rectangles represent *BARD1* exons and are numbered below. Vertical dashed lines in exon 1 and 11 represent the start and stop codons respectively. Labeled half arrows above rectangles represent forward and reverse primers used for RT-PCR. Primer pairs are labeled by integer. Forward/reverse primers are denoted by a/b respectively. (b) Illustration in (a) zoomed to highlight the region amplified by primer pair 1 (top). Gel image of resultant RT-PCR on bottom left. 3 possible products are identified and the splicing events required to yield the respective product are depicted (right).

Beyond the double mutants, we also utilized BARD1 mRNA abundance measurements to investigate variant effects on *BARD1* splicing and expression more broadly. Since alternative splice isoforms for *BARD1* have been reported^52^, we first investigated *BARD1* splicing in HAP1 to determine if these could affect variant RNA abundance. RT-PCR on wild type *BARD1* RNA detected two previously identified alternative splice isoforms in low abundance: one lacking exon 3 and another lacking both exons 2 and 3^52^(Extended Data Fig. 8a–b). These alternate transcripts are unlikely to produce functional BARD1, as both transcripts are out of frame and would likely be degraded by nonsense-mediated decay. Given their low abundance, we did not expect these alternate transcripts to significantly impact RNA abundance measurements for variants in exons 2 and 3. Thus, we designed primers to reverse transcribe, amplify, and sequence each edited exon from the canonical transcript (MANE transcript ENST00000260947.9) to identify and count variants. RNA scores were generated by taking the log_2_ ratio of the variant’s RNA abundance and genomic DNA abundance (Fig. 1a, Fig. 5c, and Extended Data Fig. 7).

26 functionally abnormal missense variants had low mRNA abundance, suggesting that these variants compromise BARD1 function at the mRNA level by altering splicing. 62% (16 of 26) were predicted by SpliceAI^53^ (SpliceAI delta score >= 0.2) reinforcing this conclusion. Additionally, three synonymous variants, c.909G>T (p.Val303=), c.1284T>A (p.Gly428=), and c.1830A>T (p.Pro610=), were classified as LoF based on SGE functional scores and also had low mRNA abundance (Fig. 5d). Despite being located away from exon-intron junctions, SpliceAI predicted high splice disruption likelihood: donor gain delta score 0.72 for p.V303=, donor gain delta score 0.85 for p.Gly428=, and acceptor gain delta score 0.99 for p.Pro610=. Notably, p.Gly428= has been identified in the human population at low frequency^43^. Similarly, we identified eight intronic SNVs with LoF functional scores (**Supplementary File 1**). Of these, five variants were predicted by SpliceAI to disrupt splicing.

Together, these results represent new insights into the expression and translation of *BARD1*. We demonstrated that BARD1 translation can successfully be started without the annotated start codon and identified SNVs that disrupt proper splicing of the *BARD1* transcript. These results highlight that the nucleotide level resolution of SGE and direct genome editing have the power to accurately detect variant effects at the level of RNA expression and protein function.

### SGE resolves missense loss of function mechanisms

Having validated that the variant effects measured by SGE are relevant to BARD1’s function as a tumor suppressor, we sought to rationalize the impact of missense variants in BARD1 on the basis of known structural features. Here we investigated sequence-function relationships across BARD1’s RING, ARD, BRCT domains and compared them to the BRCA1’s RING and BRCT.

**Extended Data Figure 9.**
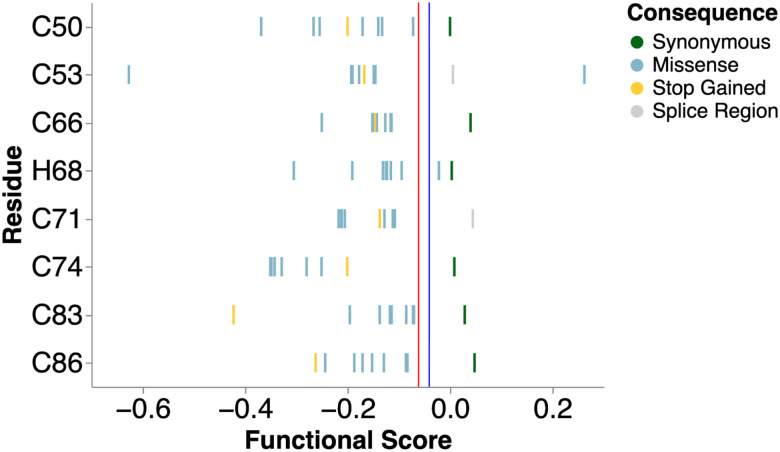
Functional scores for missense variants at Zn^2+^ binding residues Strip plot of functional scores (X-axis) for all variants at the C3HC4 residues (Y-axis) colored by indicated mutational consequence (n = 57). Each Cys and His residue position is labeled. Vertical red and blue lines indicate functional class thresholds.

**Figure 6.**
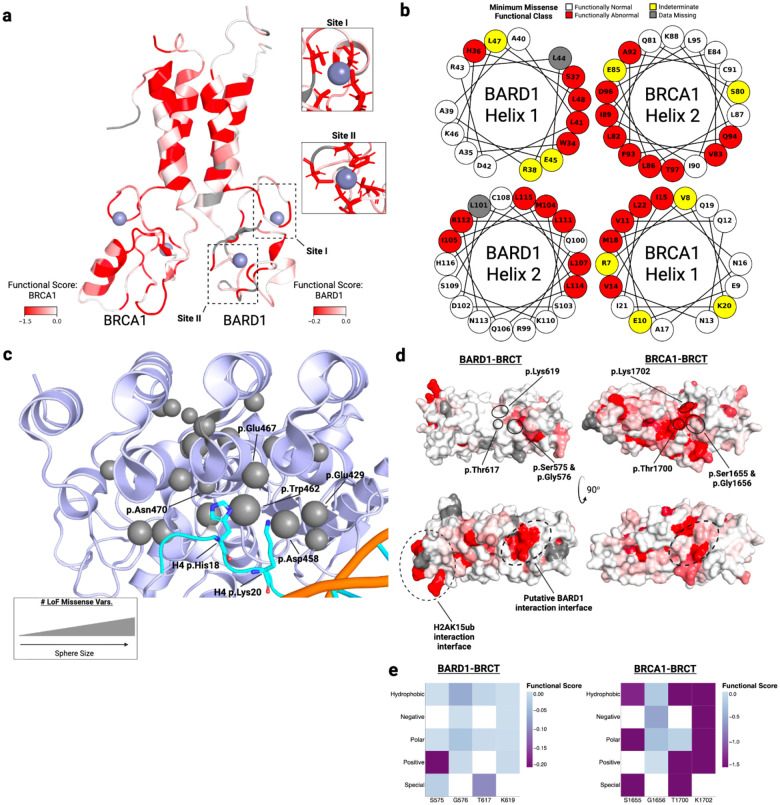
Structural consequences of BARD1 missense variants (a) NMR structure of the BRCA1-BARD1 RING:RING dimer (PDB 1JM7^54^) colored by minimum missense SGE score for BARD1 or BRCA1^25,27^. Residues without functional scores are colored gray. Residues in BARD1’s two zinc coordinating sites are highlighted. (b) The functional classes for variants in the 4-helix bundle of the RING domain are depicted as a helical wheel diagram. (c) Crystal structure of BARD1 ARD and BRCT domains bound to the nucleosome core particle (NCP) and histone H4 (PDB 7LYC^19^) zoomed in to focus on the interaction between BARD1 ARD and Histone H4 tail (cyan). Interacting residues are labeled. Size of gray spheres denote the number of LoF missense variants at each ARD residue. Positions with fewer than two LoF missense variants or with LoF proline substitutions were omitted. (d) Space-filling model of the crystal structures of the BARD1 BRCT (PDB 3FA2^67^, left) and the BRCA1 BRCT (PDB 1T29^68^, right) colored by median SGE score. Top orientation highlights the missense sensitivity of a lysine residue in the phosphopeptide binding site of both proteins. The bottom orientation highlights the H2AK15ub interaction interface of BARD1 and a missense sensitive surface on BARD1’s BRCT, hypothesized to be an interaction interface. (e) Heatmaps of median functional scores for residues in BARD1’s phosphopeptide binding site (left) and BRCA1’s phosphopeptide binding site (right) by the type of amino acid introduced. Functional scores are colored for each protein as indicated.

The structurally similar BRCA1 and BARD1 RING domains showed comparable sensitivity to missense changes with 21.3%^25,27^ and 20.1% of assayed missense variants classified as LoF respectively (p = 0.4, Fisher’s exact test). The BARD1 RING showed high missense sensitivity at functionally critical positions: 55 of 57 missense variants at the eight C3HC4 Zn^2+^-coordinating residues were LoF (Fig. 6a and Extended Data Fig. 9), and residues forming the interior of the 4-helix bundle BRCA1-BARD1 interface were also intolerant to substitution^54^ (Fig. 6b), consistent with disruption of this critical interaction.

**Extended Data Figure 10.**
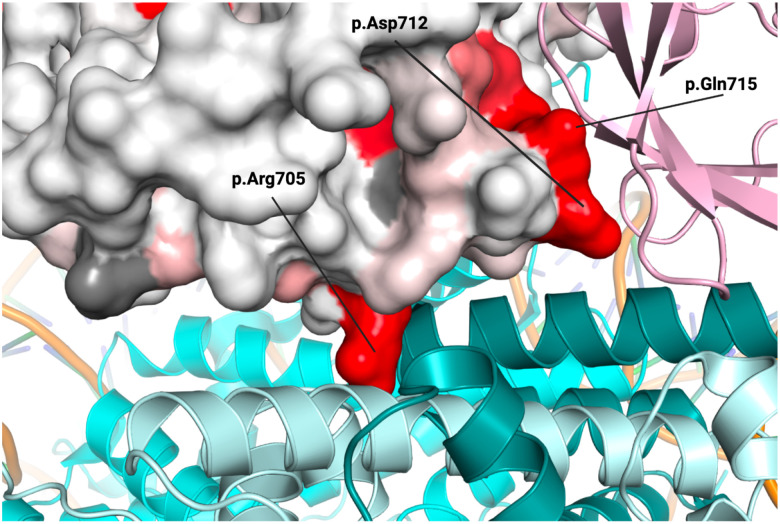
Consequences of BARD1 missense variants on BARD1 BRCT interactions Crystal structure of BARD1 ARD and BRCT domains bound to the nucleosome core particle (NCP) and histone H4 (PDB 7LYC^19^) zoomed in to focus on the interactions between the BRCT, histones H2A/H2B (light blue/dark teal) and ubiquitin (pink). A space-filling model represents the BRCT with the surface colored by mean missense score. Residues reported to be interacting are labeled.

The BARD1 ARD recognizes H4K20me0^18–20^, a marker of newly replicated chromatin that determines the cellular choice between NHEJ and HDR, while the BRCT enables rapid recruitment of the BRCA1-BARD1 dimer to dsDNA breaks^3,55^. Mapping minimum missense functional scores onto the BARD1 ARD-BRCT-nucleosome core particle structure revealed residues required for the ARD’s interaction with the H4 tail. Residues forming an acidic pocket near H4K20me0 (p.Glu429, p.Asp458, and p.Glu467) and residues forming a pocket for histone H4 p.His18 (p.Trp462, p.Glu467, p.Asn470, and p.Asp500) were highly missense sensitive and did not tolerate substitutions introducing positive charge (Fig. 6c). Like the ARD, the BRCT also binds nucleosomal histones, interacting with histones H2A and H2B^16^. Missense variants disrupting BRCT-nucleosome interface residues p.Arg705 (H2A interaction) and p.Asp712 (H2B interaction) were not tolerated (Extended Data Fig. 10). Moreover, the BRCT uniquely recognizes DNA damage-dependent H2AK13ub/K15ub marks^16^. p.Gln715, which contacts Ub p.Thr66, showed the highest missense sensitivity (Fig. 6d and Extended Data Fig. 10). Comparison of SGE results to these structures suggests that cell fitness and tumor suppression depend on BARD1’s ability to interact with the nucleosome and read H4K20me0 in newly replicated chromatin and DNA-damage-induced H2AK13ub/K15ub marks.

Furthermore, the BRCT contains a phosphopeptide binding motif consisting of surface residues p.Ser575, p.Gly576, p.Thr617, and p.Lys619^55^. We found that missense substitutions at this motif introducing hydrophobic or negatively charged residues were tolerated—contradicting the hypothesis that BARD1 requires phosphopeptide binding for HDR^19,55^ (Fig. 6d–e). In contrast, the analogous motif in BRCA1’s BRCT (p.Ser1655, p.Gly1656, p.Thr1700, p.Lys1702), which binds a BACH1 phosphopeptide^56,57^, were intolerant to charge-disrupting variants during SGE^25,27^. This differential sensitivity indicates that unlike BRCA1, the function of BARD1 that supports cell survival is phosphopeptide-independent.

Moreover, this motif has been reported to bind polyA ribose (PAR) for BARD1’s role in stalled fork protection (SFP) and recruitment to sites of DNA damage^19,55,58^. However, tolerance of charge disrupting missense variants, such as p.Lys619Glu, demonstrates that PAR binding is dispensable for essential BARD1 function in HAP1 cell survival, suggesting that BARD1’s HDR function alone is sufficient for cell survival. With SGE able to accurately predict cancer risk (Fig. 3b) and enrichment of LoF BARD1 missense variants in breast cancer cases (Fig. 3d), we provide additional evidence that BRCA1 and BARD1’s role in HDR is required for tumor suppression in humans. This finding agrees with previous studies in mice where the murine equivalent to p.Lys619 was dispensable for HDR and tumor suppression in mice^20,58,59^.

Comparing the mutational impacts on BARD1 and BRCA1’s BRCT domains revealed comparable missense sensitivity (23.2% vs. 22.3% LoF^25,27^, p = 0.93), consistent with their similar secondary structure. Despite similar structure, protein-specific variant impacts were seen. Beyond differential missense sensitivity at phosphopeptide binding sites, we identified a missense-sensitive, acidic surface on BARD1’s BRCT (residues p.Glu648, p.Glu649, and p.Glu665) that was absent in the corresponding region of BRCA1’s BRCT (Fig. 6d). We hypothesized this surface is a binding interface for an unidentified, BARD1-specific interactor required for HDR. However, examination of predicted BARD1 interactions^60^ and attempts to dock BARD1 interacting proteins^13^ using AlphaFold-Multimer^61^ did not yield any credible interactions. Future work is required to investigate the role of this pocket in BARD1 function.

All together, our identification of functionally critical BARD1 residues demonstrates that BARD1’s role in HDR is critical for tumor suppression, highlighting SGE’s ability to link specific cellular functions to disease.

## Discussion

Here we applied SGE to measure the impact of 95% of all possible SNVs and 3-bp deletions in BARD1 on cell survival and RNA expression. SGE discriminated clearly damaging variants, such as nonsense and canonical splice variants, from likely benign variants such as synonymous and intronic changes. Importantly, SGE enabled functional classification of variants with uncertain effects: we could assess whether missense and splice region variants behave like loss-of-function alleles or like functionally normal variants, providing critical evidence for clinical variant classification.

To further validate that our SGE assay captures disease-relevant biology, we demonstrated that LoF missense variants overall, as well as LoF missense variants within the ARD and the BRCT domain, are significantly enriched in breast cancer cases compared to controls. This enrichment provides orthogonal evidence that SGE in haploid cells measures phenotypes relevant to *BARD1*-associated cancer predisposition. The choice of HAP1 cells is further supported by recent work comparing *BRCA1* SGE data in HAP1 cells (myeloid origin) versus human mammary epithelial cells^27^ (HMEC). The results indicate that HAP1-derived functional scores more accurately predict variant effects in human cancer than HMEC-derived data, possibly because haploid HAP1 cells are uniquely sensitive to loss of BRCA1-BARD1-mediated HDR activity.

The high positive and negative predictive values of our SGE results for correctly identifying known pathogenic and benign controls enabled calibration to strong evidence levels for both pathogenic and benign classifications using the OddsPath framework^47^. However, achieving strong evidence calibration towards benign classifications required including nonsense variants in the pathogenic truth set, as ClinVar contains only one bonafide pathogenic missense variant in *BARD1*. While calibration using only missense controls would be ideal, the paucity of established pathogenic missense variants in *BARD1* makes this impractical. Importantly, our ability to show that LoF missense variants and nonsense variants were enriched similarly in breast cancer case-control cohorts demonstrates that both variant types confer similar disease risk, supporting this decision. Moreover, this approach—using nonsense variants for validation and calibration in genes where the pathogenic mechanism is loss of function—has established precedent in *BAP1*^30^, *BRCA1*^62^, and *RAD51C*^32^.

LoF missense variants were predominantly confined to the three known functional domains (RING, ARD, and BRCT), consistent with both their established roles in BARD1 function and results from orthogonal functional assays. However, the comprehensive nature of SGE revealed important exceptions that challenge structure-function models. Our programmed 3-bp deletion scan showed that almost no single amino acid in the intrinsically disordered domain encoded by exon 4 appears absolutely required for BARD1 function, suggesting considerable structural flexibility. In contrast, the missense variant, p.Lys140Asn, previously shown to be critical for catalyzing end resection^63^ and RAD51 loading to promote HDR^38^, scored as LoF (c.420C>G) or indeterminate (c.420C>A), in our assay despite the tolerance of most individual 3bp deletions in this region. This finding suggests that a number of positively charged residues and local flexibility, rather than most specific residues, are the key functional requirements. Supporting this interpretation, both AlphaMissense^39^ and REVEL^41^ predict mutational sensitivity in this region, likely reflecting evolutionary conservation in the underlying multiple sequence alignments.

The systematic nature of SGE also revealed that start loss variants and nonsense variants upstream of p.Met26 retained function, indicating that the annotated start codon, p.Met1, is dispensable. Similar alternative start codon usage has been observed in SGE studies of *VHL*^29^ and *RAD51C*^32^, but here, our double mutant experiments provide definitive evidence that either the canonical p.Met1 or the downstream p.Met26 can independently support full BARD1 function and normal RNA expression. These findings have direct clinical implications: it is highly likely that start-loss variants at p.Met1 that are currently classified as VUS and the nonsense variant p.Glu19Ter, currently classified as likely pathogenic in ClinVar, are benign. In fact, we have reclassified a start-loss VUS c.3G>A and p.Glu19Ter, which was also in Ambry’s dataset, to LB given our functional evidence (**Supplementary Table 2**).

Ultimately, we have generated strong functional evidence for the pathogenicity and benignity for 4,569 missense variants and show that LoF *BARD1* variants increase cancer risk. This allows for the reclassification of 95.4% (1,382 of 1,488) *BARD1* variants for which we could apply functional evidence representing 87.6% of the VUS catalogued by Ambry Genetics. In addition to hereditary breast and ovarian cancer, LoF *BARD1* variants have recently been associated with neuroblastoma^64^. For neuroblastoma patients with LoF *BARD1* variants, PARP inhibition has been shown to be an effective therapy^11^. Thus, our study immediately increased the utility of clinical genetic testing targeting *BARD1*—both in its ability to inform patients on their cancer risk and to guide treatment for cancer patients.

## Methods

### sgRNA design and cloning

All sgRNAs were designed using the default settings for the “CRISPR” function within Benchling. Off and on-target scores for all guides were generated using GRCh38 (hg38). Guides were selected based on the following criteria: ability to make a synonymous change to the protospacer/PAM, minimal predicted off-target effect, and maximum predicted on-target effect. For synonymous changes to the PAM site, C>G and G>C changes were most preferred. If no guides had PAM sites amenable to a synonymous change, two synonymous changes were made in the sequence under the guide to prevent recutting.

All sgRNAs were ordered from Integrated DNA Technologies (IDT) and cloned according to the protocol deposited here: http://dx.doi.org/10.17504/protocols.io.3byl46ddjgo5/v1.

sgRNAs were Sanger sequenced for sequence confirmation. Sequence-verified plasmids were propagated overnight in 150 mL LB and purified using the ZymoPure II Maxiprep kit and the EndoZero spin column (Zymo Research) (**Supplementary File 2**).

### Repair template library design and cloning

The sequence for the SGE target (~120 bp) was taken from hg38 and the MANE select transcript ENST00000260947.9. Fixed edits were introduced to block recutting at the sgRNA binding site. For all targets, if possible, a second synonymous substitution was also added at an alternative CRISPR cut site to allow for use of an alternate sgRNA if necessary. If this was not possible, a second synonymous substitution was made on the opposite side of the target sequence or within an intronic region to mark successful HDR editing. The sequence of the target and fixed edits was used as the template to program all possible single-nucleotide substitutions and 3bp deletions. 3bp deletions started at every nucleotide, therefore, some span one or two codons. To design the double mutant library, the target sequence and fixed edits for library 1A was used as the original template. At the canonical start codon, we retained the original sequence or introduced one of six alternate codons. These seven template sequences were then used to program all possible single-nucleotide substitutions spanning bases c.74 to c.80 of the *BARD1* transcript to generate the final double mutant repair templates. For all targets, an additional ~20 bp upstream and downstream of the sequence were obtained from the reference genome and appended to act as PCR handles.

The programmed sequences were ordered as pooled oligonucleotides and array synthesized (Twist) with the double mutant library ordered individually as an oligonucleotide pool from IDT. Oligonucleotides corresponding to a specific target were amplified using qPCR from the pool (Kapa HiFi, Kapa Biosystems) using primers specific to the PCR handle for each target. PCR products were removed from the thermocycler before plateau and cleaned using Sera-Mag Select beads (Cytiva).

Homology arms for each target were PCR amplified (Kapa HiFi) from wild-type, HAP1 gDNA using primers designed to yield homology arms that were 1,000 to 1,500 bp upstream and downstream of the region of interest. A subsequent PCR added adaptors for Gibson Assembly into pUC19 cut with EcoRI (New England Biolabs, NEB) and SalI (NEB). The homology arms and pUC19 plasmid were assembled using NEBuilder HiFi Assembly (NEB), transformed into Stellar Competent Cells (Takara), and plated on media containing ampicillin. Colonies were mini-prepped (NEB), and sequence confirmed (Plasmidsaurus). To assemble the homology arms with the repair template library amplified from the pooled oligonucleotides, sequence-confirmed homology arm pUC19 plasmids were linearized using region specific primers by inverse PCR (Kapa HiFi), digested with DpnI (NEB), and gel extracted (NEB) prior to assembly using NEBuilder HiFi Assembly (NEB). Assembly reactions were cleaned using Clean and Concentrator (Zymo Research) and were transformed into electrocompetent, 10-beta cells (NEB). After transformation, 1% was plated on ampicillin plates to assess efficiency. We targeted a minimum of 10,000 colonies, representing approximately 20X coverage of the library. The remaining 99% was used to start a 150 mL overnight culture. Libraries were then purified from the 150 mL culture using the ZymoPure Maxiprep Kit with optional EndoZero spin column (Zymo Research) and sequence confirmed.

### Cell culture and time point sampling

Cas9-HAP1 Δlig4 cells were obtained from Horizon Discovery. Cells were cultured in Iscove’s Modified Dulbecco’s Medium (IMDM) (Gibco) supplemented with 10% fetal bovine serum (MilliporeSigma). For all SGE experiments, cells were routinely sorted for 1N haploid population^25^. Sorted cells were expanded and frozen until use.

For each SGE experiment, 20 million cells were thawed into four 15 cm plates (Genesee Scientific) five days prior to transfection at a density of 5 million cells per plate. Cells were resuspended into 25 mL of media containing 10 μg/mL of blasticidin (Gibco) to select for cells actively expressing Cas9. Four days prior to transfection, the blasticidin-containing media was refreshed. Two days prior to transfection, cells were taken off of blasticidin. One day prior to transfection, cells were seeded into 10 cm plates (Genesee Scientific) at a density of 8 million cells per plate. Each plate was used for one transfection the following day.

Cells were transfected using Xfect (Takara). On the day of transfection, the media on all plates was refreshed 1 hour prior to transfection. 1 to 2 hours prior to transfection, new Xfect reaction buffer and polymer were removed from −20°C and placed at room temperature to thaw. For transfection, 12 μg of sgRNA and 3 μg of HDR library were used. For negative control transfections, 3 μg of HDR library was used but with 12 μg of sgRNA targeting housekeeping gene *HPRT1*. For each SGE region, 7 total transfection reactions were prepared in 1.5 mL tubes: 1 negative control and 6 replicates. Appropriate volumes of sgRNA and library were added, vortexed for 5 seconds, and spun down. A volume of Xfect reaction buffer was then added to bring the total reaction volume to ~750 μL prior to vortexing again. 9 μL of Xfect polymer was added to each reaction. The reaction was vortexed for 30 seconds, spun down, and incubated at 20°C for 15 minutes. After incubation, each reaction was added in a dropwise fashion to a 10 cm plate. After adding the reaction, plates were moved North, South, East, and West 2 times to distribute the transfection reagent. The 10 cm plates were incubated at 37°C for at least 4 hours. After 4 hours, the transfection reagent-containing media was aspirated and fresh media added after 2× 10 mL washes with phosphate buffered saline (PBS) (Gibco). Plates were incubated overnight at 37°C.

1 and 2 days after transfection, the media was swapped for media containing 10 μg/mL blasticidin and 3 μg/mL puromycin to select for cells actively expressing Cas9 and successfully transfected with the sgRNA plasmid. Blasticidin and puromycin media were removed 3 days after transfection and replaced with antibiotic-free media.

Days 5, 9, and 13 after transfection, cells were passaged and sampled. Samples taken on days 5 and 13 were used for sequencing. On day 5, plates were aspirated, washed with 10 mL PBS and dissociated using 2 mL, 0.25% Trypsin (Gibco). At this step two transfection plates were combined to yield 3 final replicates. 80% was collected for DNA and RNA extraction and 20% was replated in antibiotic free media and incubated at 37°C until day 9. For some regions on day 5, there were not enough cells to sample for sequencing. These experiments were repeated with 9 total transfections, where three transfection plates were combined to yield three final replicates on day 5. Additionally, during sampling of these regions, only 50% was collected for DNA and RNA extraction and 50% replated in antibiotic free media to be incubated at 37°C until day 9. On days 9 and 13, cells were sampled and passaged with 20% of cells reseeded for later time points and 80% of cells sampled.

### Sequencing preparation and sequencing

gDNA and RNA were isolated from day 5 cell pellets using an AllPrep DNA/RNA kit (Qiagen). For downstream sequencing preparation, gDNA and RNA yields were quantified using Qubit 1x Broad Range Kit (Invitrogen) and NanodropOne (Invitrogen) respectively. Only gDNA was extracted from day 13 pellets using a DNeasy kit (Qiagen). gDNA yield was quantified using Qubit 1x Broad Range Kit (Invitrogen).

Samples were prepared for sequencing by 3 sequential PCR reactions. For the first PCR, eight 50 μL PCR reactions were prepared for each replicate of each day 5 sample and 16 for each replicate of each day 13 sample; all reactions contained 250 ng of template. PCR was performed (Kapa HiFi) using the minimum number of cycles as determined from a previous qPCR reaction using wild-type HAP1 gDNA as template. Primer pairs used were designed for each target such that one primer annealed outside of the homology arm and one within the homology arm. This allowed for selective amplification from extracted HAP1 gDNA and not residual library plasmid background. After amplification, 10 μL of the respective reactions for each sample were pooled into a 1.5 mL tube and cleaned using Sera-Mag Select beads (Cytiva).

Cleaned PCR products were used in a subsequent qPCR reaction to add Illumina specific adaptors. Preparation of repair template libraries for sequencing also began at this step with 2 ng of the plasmid repair template library used instead of the previous PCR product. qPCR products were then cleaned by Sera-Mag Select beads (Cytiva) and a final qPCR was completed to add sample-specific indexes prior to sequencing. All qPCR reactions were monitored and reactions were removed from the thermal cycler prior to plateau.

For each sample, up to 5 μg of isolated RNA from day 5 pellets was reverse transcribed into cDNA (Superscript IV, Invitrogen) using a primer specific to *BARD1*’s 3’ UTR and treated with RNase H (NEB). Depending on the length of the exon targeted, preparation of cDNA for sequencing included either 2 or 3 qPCRs. For exons larger than ~300 bp in length, 3 qPCRs were performed due to the short read lengths of Illumina sequencing. The first qPCR selectively amplified (Kapa HiFi) the targeted exon from synthesized cDNA using primers in flanking exons. 8, 25 μL replicates of this qPCR were carried out for each sample. 5 μL from each replicate were pooled into a 1.5 mL tube and cleaned using Sera-Mag Select beads (Cytiva). In the subsequent qPCR reaction (Kapa HiFi) only the region of interest was amplified using target-specific primers (**Supplementary File 2**) that included adaptors for Illumina sequencing. This qPCR product was cleaned by Sera-Mag Select beads (Cytiva) and used for a final indexing qPCR (Kapa HiFi). For exons <300 bp in length, 2 qPCRs were performed. The first qPCR was analogous to the first qPCR for large exons; however, primers were designed to include Illumina-specific adaptors. After pooling, this qPCR product was cleaned by Sera-Mag Select beads (Cytiva) and indexed in a subsequent qPCR prior to sequencing.

All cleaned, indexed PCR products were quantified using Qubit 1x High-Sensitivity Kit (Invitrogen) and diluted to 2 nM. 2 nM dilutions for all samples were pooled and sequenced using the Illumina NextSeq2000 platform. For all samples, we attempted to get at least 2 million reads.

### Processing of sequencing data

Paired-end reads in FASTQ format were adapter-trimmed and merged with SeqPrep (version 1.3.2) with the following options: -A GGTTTGGAGCGAGATTGATAAAGT -B CTGAGCTCTCTCACAGCCATTTAG -M 0.1 -m 0.001 -q 20 -o 20. Reads containing one or more N bases were discarded. Merged reads were next mapped to target-specific reference sequences in GRCh38 coordinate space using the mem algorithm in bwa (version 0.7.17-r1188) to produce target-specific BAM files.

Counts of each programmed SNV within each replicate and at each timepoint, and within the repair template library, were extracted from valid reads in each BAM file by a custom Python script. All mismatches between the merged read sequence and the expected hg38-derived reference sequence were identified. Reads were considered valid if they had the expected length, contained all fixed edits, and had a single additional mismatch within the edited region of the target, which was taken as the programmed edit. Additional mismatches within the merged read but falling outside of the edited region were tolerated. The stringent read-based filtering was relaxed for SGE targets containing homopolymer runs of at least four nucleotides. In such cases, a change in sequenced homopolymer length of up to two was allowed for a read to be considered valid.

Genomic coordinates harboring a known or suspected cell line variant in HAP1 cells were discarded from analysis. Any programmed variants with fewer than ten observations in the variant library or any of the replicate and timepoint libraries were excluded from analysis.

Counts of each programmed 3bp deletion within each replicate and at each timepoint, and within the variant library, were similarly extracted from BAM files by a custom Python script. CIGAR strings were parsed to identify alignments containing a single 3bp deletion in the merged read sequence. Reads containing such deletions were further examined to ensure that all required edits were present, except for those that overlapped with the identified, programmed deletion. Reads containing sequence-level mismatches within the edited region as well as a single programmed deletion were discarded; sequence mismatches outside of the edited region were tolerated.

Each variant’s count within each replicate and at each timepoint was further converted to a variant frequency by dividing the integer count by the total number of valid reads within each dataset.

The rate at which libraries were successfully edited was calculated per replicate of each SGE target by dividing the sum of usable SNV reads and usable deletion reads by the total number of reads received. “Usable” SNV reads are defined as any read that contains all required fixed edits and an additional SNV. “Usable” deletion reads are defined as reads that contain a 3bp deletion and all required fixed edits, except for variants where the deletion overlaps with a required edit.

### Quality control

Several quality control metrics were computed for all sequenced libraries associated with each SGE target. For the starting library, at least 500,000 merged reads were required, of which at least 30% were required to be valid and no more than 10% could be wild-type. The negative control library was required to contain no more than 1% valid reads. Each replicate sequencing library at day 5 and day 13 was required to have at least 150,000 valid reads. At least two valid replicates were required for each timepoint. Finally, the Pearson correlation coefficient was calculated between each pair of replicates within each timepoint, and a minimum value of no less than 0.5 was required. Library 4J failed quality control and was removed from the dataset.

### Smoothing

A frequency ratio was computed for each variant in each day 5 replicate, comprising the log_2_ of its frequency at day 5 over its frequency in the initial library. A one-dimensional LOESS smoother, implemented in Python with the loess^65^ package (version 2.1.2) with the option span=0.20, was used to attenuate the presumed position effect on the observed variant counts owing to the location of the cut-site relative to the programmed edit. The smoother was fit separately within each replicate to the log_2_ ratios at day 5 to yield fitted log_2_ ratios. These positional fits were then subtracted from the day 5 log_2_ frequency ratios, and from each replicate’s cognate day 13 log_2_ frequency ratios, to yield adjusted log_2_ ratios. The model was fit only on those variants whose frequency at day 5 was no less than half of the starting frequency.

### Scoring

A continuous-time linear regression model, implemented in the linregress module of scipy (version 1.14.0), was used to estimate SNV and deletion scores as a function of the adjusted log_2_ frequency ratios within each replicate and each timepoint: 0, 5, and 13 days. The log_2_ variant frequency at the starting timepoint was set to 0. Estimated log_2_ fold-changes per unit time (i.e., per day) were taken as the functional score for each variant. For variants covered by two adjacent SGE targets, frequencies from both targets were jointly modeled to produce a single score. For the double mutant library, functional scores were estimated through applying a continuous negative binomial model, implemented through PyDEseq2, to variant counts at each timepoint: 0, 5, and 13 days.

RNA scores were generated for variants in the cDNA by calculating the log_2_ frequency ratio of the variant in the RNA to its respective day 5 DNA frequency. Since the genomic region sequenced in DNA samples is larger than the region sequenced in RNA samples, prior to RNA scoring, day 5 DNA frequencies were adjusted to include only positions sequenced in RNA samples. RNA scores were independently calculated for each experimental replicate and collapsed into a final score by taking the median of all replicate scores. For variants covered by two adjacent SGE targets, the final reported score is the mean of both individual scores.

### Variant annotation

The expected molecular consequence, HGVS p. identifier, and amino acid change of each programmed SNV and deletion was annotated by the Ensembl Variant Effect Predictor (vep), version 111, using the MANE Select BARD1 transcript (ENST00000260947.9). For variants with multiple predicted molecular consequences, only the consequence with the highest predicted impact was retained.

### Functional class assignment

A two-component Gaussian mixture model, implemented in scikit-learn (version 1.7.1), was fit to the SNV scores. The initial component means of the model were determined by the mean of the middle 95% of the score distribution of SNVs expected to be neutral—specifically, synonymous and intronic SNVs—and by the mean score of all nonsense SNVs outside of exons 1, 4, and 11. The probability of belonging to each fitted component was estimated for each SNV, and those whose probability of assignment to a component exceeded 0.95 were assigned that component’s label: “functionally abnormal” and “functionally normal” for the components with the lesser and greater means, respectively. The remaining SNVs were labeled “indeterminate.” The approximate score threshold values for functional class assignment were identified by taking the scores of the variants that minimized the distance to one of the 0.95 probability cutoffs. These two score thresholds were then applied to the 3bp deletion score distribution, without refitting, to yield the same three functional class categories.

Functional class categories for RNA scores were determined using the mean and standard deviation of RNA scores for nonsense SNVs. The mean and standard deviation was calculated utilizing the bottom 97.5% of RNA scores for nonsense variants (excluding exons 1, 2, and 11). The RNA score threshold for a variant to be assigned “normal” RNA abundance was set to be at least one standard deviation above the mean nonsense RNA score.

### Variant filtering

SNVs in the same codon as a fixed edit required for SGE library design or suspected cell line SNP were scored. However, with variant annotation occurring through vep using the reference *BARD1* transcript, there is a possibility of misannotation. Consequently, these variants were removed from the final data set (**Supplementary File 1**). 15 variants: c.1851G>A, c.1851G>C, c.1851G>T, c.1850G>A, c.1850G49G>C, c.1850G>T, c.1849T>A, c.1849T>C, c.1849T>G, c.1727C>A, c.1727C>G, c.1727C>T, c.1726C>A, c.1726C>G, and c.1726C>T were required for structure-function analyses in figure 6. Annotations for these variants were manually reviewed and corrected prior to analysis.

### ClinVar analysis

All single nucleotide variants and 3-bp deletions were accessed from ClinVar^8^ on September 12, 2025 and had at least 1-star review status.

### Case control analysis

Variant counts from the CARRIERS^45^ and BRIDGES^6^ breast cancer case control cohorts were received from Dr. Fergus Couch or downloaded from https://tinyurl.com/BRIDGESSummary, respectively. Variants were lifted over to HG38. The total number of patients and controls were retrieved for each cohort from their respective publications. Additionally, both cohorts reported the number of individuals from studies that did not select patients on the basis of family history (population-based) and studies that were enriched with patients with a family history of breast cancer. Odds ratios were calculated for both BRIDGES and CARRIERS cohorts individually considering all individuals and only considering individuals from population-based studies. For the CARRIERS population-based cohort only, an additional analysis was conducted subsetting for estrogen receptor (ER) negative breast cancer cases only. Training variants for the REVEL and MutPred-2 variant effect predictors were removed from the odds ratio calculation. Lastly, joint analyses were also conducted by combining both BRIDGES and CARRIERS cohorts. All odds ratios were calculated using Fischer’s Exact tests implemented in Python using scipy (version 1.13.1). P-values and 95% confidence intervals were calculated using statsmodels (version 0.14.0).

### Variant reclassification

In order to obtain the appropriate strength of evidence for the functional data, two distinct functional evidence calibrations using the current ClinGen-approved calibration method were used to generate Bayesian likelihood ratios supporting PS3/BS3 criteria^46^. First, values were derived using missense, stop-gained, splice site and synonymous variants as control sets (**Supplementary Table 2**) which corresponded to strong strength of evidence for both benign (0.0054) and pathogenic variants (316.23). Additionally, OddsPath values were recalculated using only missense variants as controls. Functional data revealed strong strength of evidence for pathogenic variants (18.5), whereas the OddsPath for benign variants was indeterminate (0.514) (**Supplementary Table 2**).

Clinically observed missense, nonsense, synonymous and intronic variants in *BARD1* were obtained from Ambry Genetics, along with publically available data on population frequency (gnomAD v4.0), *in silico* predictors (BayesDel and SpliceAI), previous functional studies^35,36^, and PVS1 annotations with thresholds applied by Ambry’s internal classification scheme for *BARD1* for variant interpretation. The dataset comprised 1,720 total variants: 1,578 variants of uncertain significance (VUS), 101 likely pathogenic or pathogenic (LP/P) variants, and 41 likely benign or benign (LB/B) variants.

For reclassification, functional data were applied to variants in conjunction with existing computational, population frequency, segregation, and de novo evidence, and 161 variants had indeterminate or no functional data (**Supplementary Table 2**). ACMG/AMP v3 points based thresholds were used for the classifications. A manual review was performed by a genetic counselor and a molecular pathologist to verify the accuracy and consistency of the initial and final variant classifications.

### External data sources

SpliceAI^53^ variant annotations were annotated by the Ensembl Variant Effect Predictor (vep). ClinVar^8^ data for germline SNVs and deletions with at least a 1-star review status were downloaded on September 12, 2025. Variants annotated as “Benign/Likely benign” were grouped with “Likely benign” variants and variants annotated as “Pathogenic/Likely pathogenic” were grouped with “Likely pathogenic” variants. Variants classified with “Conflicting interpretations of pathogenicity” were grouped with variants annotated as “Uncertain significance”. Allele frequencies for SNVs were downloaded from gnomAD^43^ v.4.1.0 on September 5th, 2024 (https://gnomad.broadinstitute.org/) and from the Regeneron Million Exome Variant browser^44^ v1.1.3 (https://www.rgc-research.regeneron.com/me/home) on August 2nd, 2024. Claude (Anthropic) was used to refine text, and figures were made using Biorender.

## Resource Availability

### Lead contact

Further information and requests for resources and reagents should be directed to and will be fulfilled by the lead contact, Lea M. Starita (lstarita@uw.edu)

### Materials availability

Please contact the corresponding author with requests for any reagents generated by this study.

### Data and code availability

SGE bioinformatics pipeline: https://github.com/bbi-lab/sge-pipeline/

Supplementary files, supplementary tables and code to regenerate all figures and analyses: https://github.com/ivanw314/BARD1_SGE_analysis

Raw sequencing reads can be downloaded from IGVF: https://data.igvf.org/analysis-sets/IGVFDS4537CHCJ/

Functional scores are available through MaveDB: https://www.mavedb.org/score-sets/urn:mavedb:00001250-a-1

## Extended Data
